# The Profession’s Interests above the Individual

**Published:** 1896-04

**Authors:** 


### THE PROFESSION'S INTERESTS, ABOVE THE INDIVIDUAL.

The subject of higher veterinary education in America is too grave and serious a one to draw down to the level of personalities. It is above any individual, and the part one plays in aiding its accomplishment can only be the discharge of one's duty as a member of his adopted profession. One's duty lies not in tearing down what has already been achieved, even though it has not been fashioned by his own plans, but commands one and all to smooth off the rough edges and direct the chief purposes of such a movement in the highest channels it deserves. One's responsibilities in this course are always to by measured by his place in the profession, and when filling high offices as directors of schools, or the higher offices of association circles, the more lofty and responsible the place the more heavily freighted are the shoulders that accept such duties. Veterinary education has progressed faster and better in America in the past ten years than at any other period, and stronger than any other profession. We lay no special claim to having aided this grand movement beyond the discharge of our duty as we interpreted it. That it has not grown stronger and more rapidly lies only with those who, not by precept but by example, have more than once checked its forward career, and for this they must some day answer. There will be no turning backward, for the sun has forever set for short-term curriculums, and a failure to realize the needs of the hour will only leave memories behind of those who sought to lead, but failed in the final test. Upward, onward, will be the alluring attraction of the Association of Faculties that has given so great an impetus to this movement. The common purpose, the single desire, the bond of fellowship in this great work, will yield the best results and faithfully discharge every responsibility that such places of honor and trust command.

---

UNDER the head of correspondence will be found a letter from Illinois, fruitful in thought and quite equally forceful in many other States. It well accounts for the lack of successful legislation of importance to the well-being of the veterinarian in that as well as other States. The need of more thorough work in the strengthening of association circles and the closer unity of efforts centrally directed are strongly pointed out. In these trying days when so many are tempted to wander away from the true fold and turn their steps into devious pathways of dishonor to our profession, and imposing upon the public by false and misleading statements, nostrums, and proprietary remedies of more than questionable value, we could cry aloud unto them: "Oh! that you would exhaust such efforts in endeavoring to raise up the standard of your calling to the higher plane that she so well deserves, and give such influences as you are wasting on selfish ends to agitating for better veterinary sanitary laws and the creation of a veterinary sanitary police, by which there would be little to-day to complain of in the lessened demand for veterinary service."

---

THE many friends of Dr. T. J. Turner, of Glasgow, Missouri, will regret to learn that, at the expiration of his term in December last as State Veterinarian of Missouri, he failed to be reappointed. Faithful in all his duties as a public servant, and never so well equipped as at this time, after a number of years of valuable experience, it would seem that in these trying times a State properly alive to its own interests would not wish to lose so valuable a servant, when his service and experience could so ill be spared. When will the great body of our intelligent people cease to worship at the altar of so-called practical politics, the infamous and pernicious spoils-system, that stops not in its bold demands at the question of the nation's, State's, or city's peril, or where is so directly involved the health and happiness of our people, so much conserved by faithful servants in positions of this kind?

---

THE JOURNAL is in receipt of several communications from the Toronto Veterinary College bearing upon the subject-matter of an editorial in the February number. In a letter from Prof. Duncan we learn that the alleged incident of Thanksgiving Day was greatly exaggerated, and from Prof. Smith a statement signed by his class representatives that the most agreeable and pleasant relations exist between the American and Canadian students.

The information published in the JOURNAL was received from a number of sources, and verified as far as possible through graduates of that school. We trust that in the future when the public press contains reports of this and of any similar nature the JOURNAL may at once be furnished with a statement from the college or the association involved, and that letters sent to the parent by her children seeking information will not go unanswered.

---

THE JOURNAL congratulates the *American Veterinary Review* on its added strength through the association therewith in joint-ownership and editorial direction of Prof. Roscoe R. Bell, of Brooklyn. With this infusion of new blood we bespeak for the *Review* a wider range of usefulness, an added power in educational channels, and a strengthening of veterinary journalism for the great work before it. The March number of the *Review*, with the wealth of "Chit-chat," was one of the most attractive numbers for several years.

---

TEN years ago the writer urged upon his fellow-alumni the crying need of their taking a stronger interest in matters political, that while thus becoming more useful citizens, better veterinarians, they would become a factor in moulding legislation which to-day presses upon them with so much force, and which finds our profession as a body so illy equipped to guide and direct our people in matters that demand immediate settlement, and which must come through legislative bodies.

THE half-million dollars lost by the State of Delaware in the last ten years, from the ravages of cerebro-spinal meningitis among her horses, wisely expended would have reared for that State the most complete veterinary sanitary police in existence, and left a large balance in the possession of her people, through saving measures that would have been adopted by such a guard, and no doubt would have contributed to our whole country beneficent measures that would have added millions to the wealth of our people on whom these serious losses have fallen.

---

THE temper and position of the veterinary profession of to-day on the subject of higher veterinary education is well shown in this number under the head of Legislation, taken from the records of the present session of the Legislature of Manitoba, and we *ask* the question, "How much longer will the short-term schools continue in the face of public opinion so strongly set against them? Why will they not bend, rather than be broken? Do they not realize how narrow the field of their sustenance daily grows?" These conditions must sooner or later be answered, and soon at the latest.

---

THE following letter was recently received by the President of the Pennsylvania State Veterinary Medical Association, and needs no comment for the dire need of a State Board of Examiners in the Keystone State. The writer of the letter is a graduate of one of the leading veterinary colleges of the United States:

Mr. W. H. Ridge, Yours Recd This Date and will Say in Reply Do Not Care For the Extra $3.00 only you Should Have Informed Me If the Doos Was $2.00 Instead of $1.00. In Regards Too The Attending whos Business Is It whither I am Present or Not. The Meetings Cannot Be verry Interesting when Their is Not a Corum Present. you Spoke of Colecting Perhaps you will. I Am No Thief Nor a Bumm, & People Do Not Have to Place accounts that I Owe in a Collectors Hands. This Is all I Have to Say If you will Ensure Me of My Name Being Erased From The Roll For All Time I will Send you a Cheque For $6.00 as I Have Never Attended The Meetings & Never will. you Claim Too Do So Much For The Profession Then Have a Law Passed that a Graduate Must Pass a Examination Before a Board of Just ordinary Practitioners An Insult too Eny and All College Faculties & Surely an Injustice too Many a yong Man who Has Spent Perhaps the Last $ For an Education, & Then Allow Non Graduates & Dude Graduates That Could Not Tell A Horse from A Cow if The Cow was Dehorned. — — ———

THE JOURNAL regrets exceedingly to learn of the impaired health of our esteemed colleague, Dr. J. H. Wattles, of Kansas City. The work and zeal of Dr. Wattles are too well known to need any encomium from the JOURNAL. The need of more faithful members of the profession like him is so great a one that we shall most earnestly hope and wish for his early restoration to health and return to the great field of usefulness he has so well filled.

---

### NEW PUBLICATIONS.

FLEMING'S VETERINARY OBSTETRICS. Second edition. From J. A. Carveth & Co., Toronto, Canada. Price, $5.50. The reaching of a second edition of this valuable work is the best commendation of its worth and the great need of such a text-book in the advancing profession of veterinary science. We shall not indulge in the disposition of some critics as to how completely original this work may be, for in such a great undertaking, dealing with fixed and well-grounded deductions in many directions, there must be of necessity great similarity; suffice it to say on this point that the growth of veterinary obstetrical science in English-speaking countries has been wonderfully aided and advanced through the first edition of this work. As a contributor to English veterinary literature, Prof. Fleming needs no introduction, for the wealth of good things from his pen is appreciated by all. The typographical work is well done, and much of the matter and illustrations in the forepart of the work are entirely new. We note the recognition of the use of impregnators in undue contraction of the os, as suggested by Dr. Lyford, of Minnesota. The value of this work will increase day by day, for the future veterinarian must be as well equipped in bovine practice as the present one is in equine and canine pathology, and the need of a thorough obstetrical knowledge is of greater moment in bovine practice than any other branch of the veterinarian's work. Every need in this direction will be conserved by a careful perusal of this work, and those who have thoroughly studied the first edition will be eager to add the second to their libraries, to gain the greatly added-to pages in the second edition.

Electricity in Electro-therapeutics. By Edwin J. Houston, Ph.D., and A. E. Kennelly, Sc.D. Published by the W. J. Johnston Company, of New York, as one of the Elementary Electro-technical Series. This is a neat little volume with an unusual amount of valuable information on this subject crowded within its pages—its purpose a highly commendable one, to offer real scientific information on this now all-important subject to the students and practitioners of medicine; offers also to the veterinarian a field of experimental study that in its already reported use by one of our Western members of the profession gives promise of filling a more important place in our measures for relief to our patients. Unfortunately this subject for many years has been in the hands of empirics and impostors who have fed upon an ignorant and gullible public that now, with a large number of highly educated practitioners pursuing its study and use, there would seem no more suitable opportunity for the issue of this work, from so eminent authors as the above, and we are sure it will meet with a generous reception.

An Illustrated Dictionary of Medicine, Biology, and Allied Sciences. By George M. Gould, A.M., M.D. Published by P. Blakiston, Son & Co., 1012 Walnut Street, Philadelphia. Every possessor of one of these storehouses of knowledge wonders how it was possible for him to get along without this work, unequalled by any of its kind in the English language. Its attainment of a third edition speaks volumes for its worth; how great a need for such a work in these rapidly advancing times, and how quickly the up-to-date scholar discerns works of great merit. In opening its pages for the first time our attention was riveted on the subject of parasites, and of so much interest did we find this subject treated, to a student of comparative science, that its wealth of information seemed rather that of an encyclopædia than a dictionary. The progressive veterinarian will find it an adjunct to his library that he can no longer deny himself, and we hope that in a future edition it may extend itself to the field of the veterinarian in a collation of many terms that are purely veterinary in derivation and meaning. All who peruse its pages will acknowledge a debt of gratitude to the author for the labors entailed in its compilation.

